# Predictors of Fracture Risk and Bone Mineral Density in Men with Prostate Cancer on Androgen Deprivation Therapy

**DOI:** 10.4061/2011/924595

**Published:** 2011-07-28

**Authors:** Katherine Neubecker, Beverley Adams-Huet, Irfan M. Farukhi, Rosinda C. Delapena, Ugis Gruntmanis

**Affiliations:** ^1^Department of Medicine, University of Texas, Southwestern Medical Center, Dallas, TX 75390, USA; ^2^Departments of Clinical Sciences and Medicine, University of Texas, Southwestern Medical Center, Dallas, TX 75390, USA; ^3^Department of Nuclear Medicine, Dallas Veterans Affairs Medical Center, Dallas, TX 75216, USA; ^4^Department of Medicine, Dallas Veterans Affairs Medical Center and University of Texas, Southwestern Medical Center, Dallas, TX 75216, USA

## Abstract

Decrease of bone mineral density (BMD) and fracture risk is increased in men with prostate cancer receiving androgen deprivation therapy (ADT). We looked at possible predictors of decreased BMD and increased fracture risk in men with prostate cancer; most of whom were on ADT. In a retrospective study, we analyzed serum, BMD, and clinical risk factors used in the Fracture Risk Assessment (FRAX) tool and others in 78 men with prostate cancer with reported height loss. The subjects were divided in two groups: 22 men with and 56 without vertebral fractures. 17 of the 22 men with vertebral fractures on spine X-rays did not know they had a vertebral fracture. Of those 17 men, 9 had not previously qualified for treatment based on preradiograph FRAX score calculated with BMD, and 6 based on FRAX calculated without BMD. Performing spine films increased the predictive ability of FRAX for vertebral fracture. Vertebral fracture was better predicted by FRAX for other osteoporotic fractures than FRAX for hip fractures. The inclusion of BMD in FRAX calculations did not affect the predictive ability of FRAX. The PSA level showed a positive correlation with lumbar spine BMD and accounted for about 9% of spine BMD.

## 1. Introduction

220,000 men are diagnosed with prostate cancer each year, and more than 40 percent receive androgen deprivation therapy (ADT) as initial treatment [1, 2]. ADT has been shown to decrease bone mineral density (BMD) as measured by dual-energy X-ray absorptiometry (DXA) as well as increase risk of fracture. After initiating ADT, BMD decline starts shortly after [3, 4]. A meta-analysis of almost 117,000 men demonstrated that patients on ADT had lower total BMD, 30% increased risk of osteoporosis, and 70% increased risk of fractures [5]. In a study of over 47,000 men with prostate cancer by the National Cancer Institute (NCI) and Medicare, those treated with ADT or bilateral orchiectomy within six months of diagnosis had a 5-year fracture risk of 19.4% versus 12.6% in controls matched for patient and tumor characteristics [6]. A systematic review of five studies on prostate cancer patients in the US and abroad, which included the NCI-Medicare study, found a 23% increase in fracture risk for men treated with ADT [7]. 

Fracture Risk Assessment (FRAX) is an algorithm developed by the World Health Organization to determine fracture risk of men and women [8]. The risk factors in the algorithm include age, current height, prior fracture, parental history of fracture, BMD of the femoral neck, and secondary osteoporosis risk factors such as hypogonadism. Yet, inclusion of secondary causes such as hypogonadism does not change FRAX-calculated risk of fracture when femoral neck BMD is included. The National Osteoporosis Foundation's recommended treatment thresholds based on FRAX score are 3% for hip (“Hip FRAX”) and 20% for major osteoporotic fractures (“Osteoporotic FRAX”) [9]. Prior studies of men with prostate cancer on ADT by Saylor et al. and Adler et al. [10, 11] demonstrated that the inclusion of BMD values in the FRAX calculation led to lower FRAX scores, and thus fewer people qualified for treatment. These studies also found that the majority of men who met treatment thresholds did so because of the hip FRAX rather than osteoporotic FRAX. Men younger than 70 [10] and of African-American ethnicity [11] were less likely to meet treatment thresholds. 

Prior studies have addressed factors that affect treatment qualifications in men on ADT based on FRAX scores. The utility FRAX for predicting incidence of fracture in men with prostate cancer has yet to be determined. Many of vertebral fractures are clinically unrecognized due to lack of complaints, yet, they are strong predictors of future fractures at all sites. Two and half inches or greater height loss in men is associated with increased risk of vertebral compression fractures [12]. The utility of height loss in predicting vertebral fracture in men with prostate cancer has not been established. 

## 2. Materials and Methods

### 2.1. Subjects

This was a retrospective study of 78 male patients with prostate cancer (median age 77: 55 Caucasian, 19 African-American, 3 Hispanic, and 1 Spanish) at the Dallas Veterans Affair Medical center (DVAMC) approved by the Institutional Review Board of DVAMC, Dallas, Tex, USA. 

Subjects had been referred to the Osteoporosis Clinic by the Department of Urology for management of bone health, either while receiving ADT (*N* = 63) or before initiating ADT (*N* = 15). All men at DVAMC receiving ADT or about to initiate ADT are referred by Urology Department to Osteoporosis clinic regardless of whether they have history of osteoporosis. At initial visit at Osteoporosis clinic, spine X-rays are obtained on all patients whose measured height in clinic is at least one inch less than their self-reported young adult height. Inclusion criteria were height loss and having spine X-rays, which were performed near or at the time of the initial Osteoporosis Clinic visit. Height loss was determined by the height measured at the initial Osteoporosis Clinic visit compared to self-reported young adult height. Patients with pathologic fractures thought to be secondary to bone metastases were excluded. The primary outcome of the study was whether height loss predicted fracture and BMD in men with prostate cancer on ADT. Secondary endpoints: in how many men would FRAX score change with finding compression fracture on spine X-ray, and if the duration of ADT and level of prostate-specific antigen (PSA) could predict BMD and fractures at any site. 

### 2.2. Statistical Analysis

Comparisons of subjects with and without vertebral fractures were made with the Wilcoxon Rank Sum test. Univariate associations between continuous variables were assessed with Spearman correlation coefficients. Logistic regression analysis was used to assess predictors of fractures and to estimate unadjusted and adjusted odds ratios for fracture risk. Multiple linear regression analysis was conducted to further evaluate the relationship between spine BMD as the dependent variable and PSA while controlling for age, weight, and use of vitamin D supplements. Data with positively skewed distributions were log-transformed prior to parametric analysis. Statistical analysis was performed with SAS version 9.2 (SAS Institute, Cary, NC, USA). 

To assess the utility of FRAX scores in predicting of vertebral fractures, receiver operating characteristic (ROC) curves which plot 1-specificity versus the sensitivity of the diagnostic value of the model were constructed, and the area under these curves (AUC) and 95% confidence intervals were calculated. An ROC AUC of 0.5 indicates chance performance, and an ROC AUC of 1.0 is the ideal, maximum AUC [13]. 

### 2.3. Radiography/Laboratory Measurements

BMD of the femoral neck, total hip, and lumbar spine were measured by DXA (Hologic QDR 4500A, Waltham, Mass, USA) in the DVAMC Nuclear Medicine Department. The least significant change in g/cm^2^ for regions of interest in grams per square centimeter was as follows lumbar spine 0.031, total hip 0.037, and femoral neck 0.029. Serum total testosterone was measured by electrochemiluminescence immunoassay, alkaline phosphatase by the p-nitrophenyl phosphate method, 25-hydroxy vitamin D by liquid chromatography/tandem mass spectrometry, PTH by chemiluminescent immunoassay, and calcium by indirect potentiometry. Intra-assay coefficients of variation for all of the assays were less than three percent, and interassay coefficients of variation were less than 8 percent. 

## 3. Results and Discussion

At baseline, men with prostate cancer, with or without vertebral fracture, were not statistically different, except for total testosterone levels and FRAX scores (Table 1). 

Testosterone levels between groups were statistically different (*P* = 0.01), yet, levels in both groups were profoundly low. The FRAX scores for hip fractures and other osteoporotic fractures were also statistically different (Table 2), as expected, when men with vertebral fractures and no vertebral fractures were compared. 

None of the primary outcomes were positive. The mean difference of height loss between men with and without vertebral fractures group was only −0.19 cm (95% CI: −0.83 to 0.45). Degree of height loss did not predict either bone density or fractures at any site; risk of vertebral fracture based on height loss produced an odds ratio of 1.1 (95% CI: 0.76 to 1.65). This can likely be explained by our inclusion criteria. In our clinic we order spine X-rays in men who have lost more than one inch of height, and, therefore, we could not compare men with and without height loss. 

Yet, 17 of the 22 subjects with vertebral fractures had no known history of vertebral fracture until they were discovered incidentally on spine films ordered due to a loss of height. Had these fractures not been detected by spine X-rays, nine (about 41%) of the 22 men with fractures would have not qualified for treatment based on FRAX calculated with BMD or six (about 27%) based on FRAX calculated without BMD. 

Discovery of incidental vertebral fractures on spine films increased the predictive ability for vertebral fracture of all types of FRAX scores: hip FRAX with or without BMD and osteoporotic FRAX with or without BMD (Table 4). All post-X-ray hip FRAX and osteoporotic FRAX were statistically significant (AUC significantly different from null of 0.50). Osteoporotic FRAX demonstrated greater predictive ability for vertebral fracture than hip FRAX, 0.74 versus 0.66 AUC, respectively, without BMD (*P* = 0.01) and 0.72 versus 0.66 AUC, respectively, with BMD (*P* = 0.04). The ROC models for predictive ability of FRAX were not significantly changed by including a factor accounting for whether the patient was already on ADT. Thus, data for all 78 patients (63 already on ADT, 15 not on ADT) is displayed.

**Figure 1:**
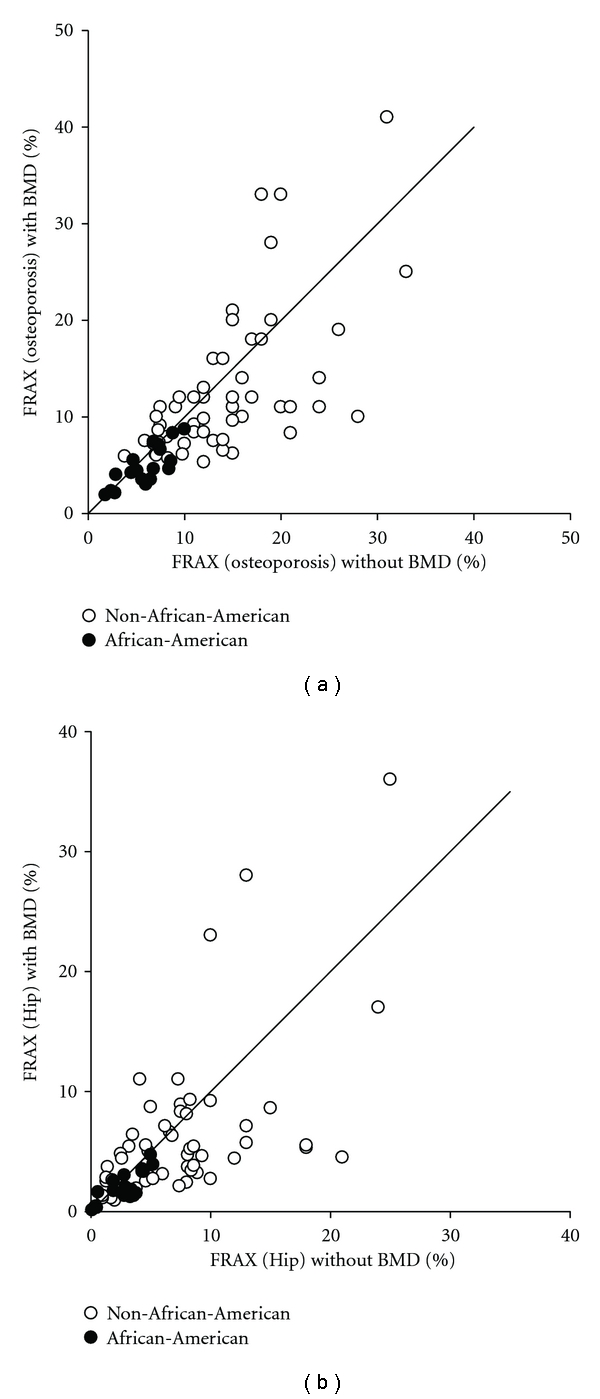


The inclusion of BMD in FRAX calculations did not affect the predictive ability of FRAX. FRAX scores calculated with and without BMD were significantly related (Table 2). Spearman correlation coefficients were rho = 0.80 (*P* < 0.001) for osteoporotic FRAX (Figure 1(a)) and rho = 0.71 for hip FRAX (*P* < 0.001) (Figure 1(b)). 

**Figure 2:**
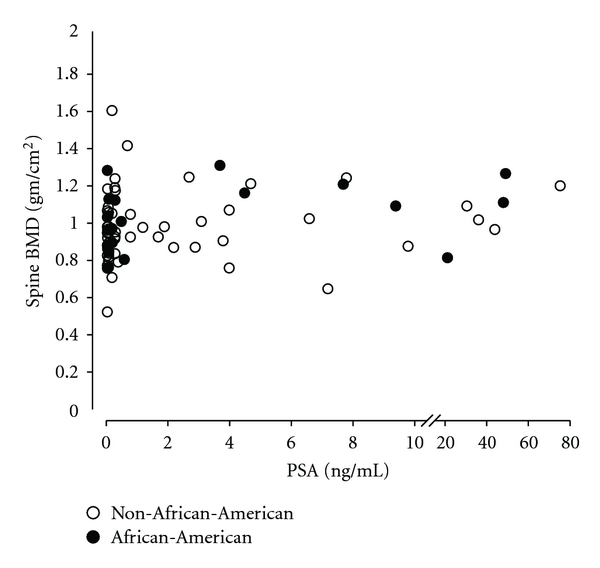


There were too few fractures in the African-American group to give adequate precision so conclusions cannot be reliably drawn from the African-American-specific data. ROC AUC appears high in AAs because of high specificity due to low incidence of vertebral fracture (Table 4). Race was not correlated with spine BMD in multivariate linear regression models controlling for age, PSA level, and use of vitamin D supplementation. The PSA level showed a positive correlation with lumbar spine BMD (Figure 2) in both the univariate linear regression model (*P* = 0.03) and the multivariate linear regression models controlling for age, weight, and use of vitamin D supplements (*P* = 0.04), as displayed in Table 3, and accounted for about 9% of spine BMD. Weight correlated with BMD in the spine (rho = 0.25, *P* = 0.03), femoral neck (rho = 0.43, *P* < 0.0001), and total hip (rho = 0.43, *P* = 0.0001) (data not shown). Age had a positive correlation with BMD at lumbar spine (*P* < 0.02) but not at femoral neck or total hip. 

From other secondary outcomes, neither duration of ADT, level of 25-hydroxyvitamin D, parathyroid hormone, total alkaline phosphatase, nor total testosterone levels predicted BMD or fractures at any site in our study population. 25-hydroxyvitamin D level was found to be below 20 ng/mL in 31% and 20–30 ng/mL in 20% of men. In the other 49% of men, 25-hydroxyvitamin D was above 30 ng/mL. 

## 4. Conclusions

The utility of height loss and FRAX scores as predictors of fractures in men with prostate cancer had not been previously studied. In our study population of older men with prostate cancer, most of whom were on ADT, height loss was similar in men with and without compression fractures and other osteoporosis-related fractures. In conclusion, our study cannot suggest a threshold of height loss for which spine X-rays or perhaps vertebral fracture assessment with DXA should be used. 

As with prior studies by Adler et al. [11] and Saylor et al. [10], fewer men met treatment criteria when BMD was included in FRAX score calculation. Also, fewer men met treatment threshold for major osteoporotic fracture risk than for hip fracture risk. Compared to Adler et al., our study population had similar age (both with median age 77 years) and weight (85.5 kg versus 88.1 kg in Adler et al.) and excluded men with fractures secondary to bone metastases (versus 30% with bone metastases in Saylor et al.). Our study population was 24% African-American population, compared to 58% in Adler et al. and 5% in Saylor et al. 

The utility of FRAX in predicting fracture incidence in men with prostate cancer had not been studied previously. An advantage of the current study is that incidence of vertebral fracture was known due to spine radiographs, so the predictive ability of FRAX scores with or without BMD, for hip versus osteoporotic, and across ethnic groups could be analyzed. Our study demonstrated that discovery of incidental vertebral fractures on routine spine films increased the predictive ability for vertebral fracture of all types of FRAX scores. Another novel finding was that vertebral fracture in men with prostate cancer was better predicted by osteoporotic FRAX than hip FRAX. The FRAX score (without knowledge of spine fractures) calculated with BMD underestimated therapeutic threshold in nine men with previously unknown spine fractures in our study. As a result we feel that most men with prostate cancer on ADT, and with any height loss, should have spine X-rays taken to estimate their future fracture risk accurately. 

It should be mentioned that hypogonadism is one of the “secondary causes” in the FRAX calculator but does not change the FRAX score if and when BMD of the femoral neck is used in calculation. We believe that patients with prostate cancer on ADT may be different from “typical” patients with hypogonadism. It is well known that patients with prostate cancer on ADT have mostly undetectable levels of testosterone and as the result have low estradiol levels. Both testosterone and estradiol are imperative in preserving bone density, quality, and in predicting the risk of future fractures. Moreover, the effects of ADT on BMD and fracture risk persist for years after ADT is completed [6]. 

The positive correlation of age and spine BMD may be an artifact of falsely elevated BMD in lumbar spine due to vertebral body degeneration since the group without vertebral fractures had lower spine T scores (higher spine BMD) than the group with vertebral fractures. The significance of the positive correlation of PSA level and lumbar spine BMD is unclear. PSA accounted for 9% of the variability in lumbar BMD. The mechanism by which PSA affects bone metabolism is not clearly defined, but it seems to regulate the biologic activity of parathyroid-hormone-related protein (PTHrP). PSA specifically cleaves PTHrP, which subsequently eliminates the ability of PTHrP to stimulate cAMP production [14]. Moreover, PSA appears to promote osteoblast differentiation via transcription factor Cbfa1 [15]. PSA prevents bone resorption by inducing osteoprotegerin activity and inhibiting the receptor activator of nuclear factor-kB ligand (RANKL) expression on osteoblasts [16]. Osteoblasts produce cytokines including IL-6 that appear to lead to androgen-independent induction of PSA genes [17]. Increases in PSA may lead to greater production of endothelin-1, a protein that stimulates osteoblasts and inhibits osteoclasts in the presence of androgen-insensitive prostate cancer cells [18]. All of the above factors may explain why spine BMD may be higher in men with elevated PSA. 

Of note, Vitamin D levels were fairly replete in both groups of men, those with and without fractures. This is likely because the Urology Clinic at the Dallas VAMC often empirically starts Calcium plus Vitamin D 500 mg/250 U replacement twice a day before we see men in the Osteoporosis clinic. 

Limitations of our study include the small sample size, retrospective data collection, and possible selection bias from inclusion criteria. Based on prior studies discussed above, one would expect BMD and fracture risk to be associated with testosterone levels and the duration of ADT. 

However, we did not find it in our study. The cross-sectional nature of the study is not optimal for evaluating predictors of fracture since fractures are already present at the time the variables are assessed. Our inclusion criteria likely generated some selection bias due to the inclusion criteria of height loss. A future study could include subjects without height loss to compare groups with or without height loss. The range of height loss was relatively small with over 50% of subjects with height loss of 2 inches or less. 

Including BMD of distal 1/3 radius has been shown to increase sensitivity of DXA scans in detecting osteoporosis [3] but is not included in routine DXA scans at DVAMC. 

Going forward it would be important to study prospectively if the FRAX calculator is a precise predictor of fractures in men with prostate cancer on ADT. A future study with a prospective approach could better determine predictors of fracture by performing spine films at two points in time to assess for new fractures. Further study in a larger sample is needed to address the predictive ability of FRAX across ethnic groups. 

## Figures and Tables

**Table 1:** Baseline characteristics stratified by presence of vertebral fracture.

	No vertebral fracture							Vertebral fracture						
	*n* = 56							*n* = 22						
Variable	Median	Mean	SD	Range				Median	Mean	SD	Range			*P* value*
Duration of ADT (months)	16.5	39.6	44.7	0.0–10.5				10.5	30.3	43.5	0.0–156.0			0.21
Age (years)	77.0	76.5	8.2	58.0–77.5				77.5	78.0	6.7	63.0–88.0			0.53
Weight (kg)	83.5	84.8	17.1	54.5–86.7				86.7	87.1	18.0	54.8–119.8			0.46
Height (cm)	172.7	171.7	6.1	152.4–171.4				171.4	172.5	7.1	157.5–182.9			0.83
Height loss (cm)	5.1	5.8	3.0	0.8–5.8				5.8	6.4	3.6	1.3–14.0			0.58
BMI (kg/m^2^)	28.4	28.5	4.9	18.2–28.6				28.6	28.7	5.5	18.3–40.4			0.76
25-(OH) Vitamin D (ng/mL)	31.1	30.3	14.6	7.0–26.1				26.1	27.1	10.9	8.0–57.8			0.42
Serum calcium (mg/dL)	9.7	9.5	1.3	0.7–9.6				9.6	9.5	0.5	7.9–10.6			0.52
Parathyroid hormone (pg/mL)	44.0	49.5	28.1	16.7–37.3				37.3	45.9	28.8	15.8–135.0			0.49
Alkaline phosphatase (U/L)	80.0	92.4	80.6	49.0–73.0				73.0	74.2	17.0	47.0–112.0			0.16
Prostate-specific antigen (PSA) (ng/mL)	0.3	6.2	15.1	0.1–0.3				0.3	2.8	6.7	0.1–30.7			0.49
Testosterone (ng/mL)	0.1	0.6	1.3	0.1–0.2				0.2	1.4	1.7	0.1–5.9			0.01

*P values are from the Wilcoxon rank sum test.

**Table 2:** BMD and FRAX stratified by presence of vertebral fracture.

	No vertebral fracture							Vertebral fracture						
	*n* = 56							*n* = 22						
Variable	Median	Mean	SD	Range				Median	Mean	SD	Range			*P*-value*
BMD, femoral neck	0.7	0.7	0.1	0.5–1.0				0.7	0.7	0.1	0.5–0.9			0.34
T score, femoral neck	−1.8	−1.9	0.7	−3.3–−0.7				−2.2	−2.0	0.8	−3.4–−0.6			0.33
BMD, total hip	0.9	0.9	0.1	0.6–1.2				0.8	0.8	0.2	0.5–1.2			0.22
T score, total hip	−1.2	−1.3	0.9	−3.0–0.3				−1.4	−1.5	1.1	−3.7–0.0			0.51
BMD, lumbar spine	1.0	1.0	0.2	0.8–1.6				0.9	0.9	0.2	0.5–1.3			0.11
T score, lumbar spine	−1.1	−0.9	1.6	−4.0–4.6				−1.8	−1.6	1.8	−5.2–2.1			0.10
FRAX (Osteo) without BMD (%)	9.3	10.8	6.2	1.8–33.0				15.0	16.0	6.8	6.8–31.0			0.001
FRAX (Hip) without BMD (%)	4.0	5.5	4.8	0.1–24.0				6.8	8.1	6.0	1.7–25.0			0.03
FRAX (Osteo) with BMD (%)	7.5	9.5	6.9	1.9–33.0				11.0	14.0	7.8	5.4–41.0			0.002
FRAX (Hip) with BMD (%)	3.0	4.5	5.1	0.1–28.0				4.5	6.6	7.1	1.1–36.0			0.03

*P values are from the Wilcoxon rank sum test.

BMD measurements are in g/cm^2^.

**Table 3:** Summary of multiple regression analysis for variables predicting spine bone mineral density.

	Variable	PSA*	Age	Weight	Vitamin D supplement	Race	*R* ^2^
Model 1	*B (SE)*	0.02 (0.01)	—	—	—	—	0.06
	*P*	0.03	—	—	—	—	

Model 2	*B (SE)*	0.02 (0.01)	0.005 (0.003)	—	—	—	0.08
	*P*	0.05	0.08	—	—	—	

Model 3	*B (SE)*	0.02 (0.01)	0.005 (0.003)	0.0007 (0.0005)	—	—	0.09
	*P*	0.05	0.04	0.17	—	—	

Model 4	*B (SE)*	0.02 (0.01)	0.004 (0.002)	—	−0.13 (0.04)	—	0.22
	*P*	0.045	0.12	—	0.001	—	

Model 5	*B (SE)*	0.02 (0.01)	0.004 (0.002)	—	−0.12 (0.04)	0.04 (0.04)	0.23
	*P*	0.05	0.13	—	0.001	0.33	

Model 6	*B (SE)*	0.02 (0.01)	0.004 (0.003)	0.0004 (0.0005)	−0.12 (0.04)	—	0.23
	*P*	0.04	0.04	0.40	0.003	—	

B = regression coefficient, SE = standard error.

*log _e_ transformed PSA.

Race is modeled as African-American versus non-African-American.

**Table 4:** FRAX score in the prediction of vertebral fracture in men with prostate cancer.

		All Subjects		Non-African-American		African-American	
		(*N* = 78; vertebral		(*N* = 59; vertebral		(*N* = 19; vertebral	
		Fx = 22/78 = 24.4%)		Fx = 18/59 = 31%)		Fx = 4/19 = 21%)	
		ROC AUC	95% CI	ROC AUC	95% CI	ROC AUC	95% CI
FRAX (Hip) without BMD	before X-ray	0.55	0.39–0.70	0.59	0.42–0.77	0.42	0.15–0.70
	after X-ray	0.66	0.53–0.78	0.63	0.47–0.78	0.72	0.42–1.00

FRAX (Osteo) without BMD	before X-ray	0.59	0.43–0.75	0.66	0.48–0.84	0.50	0.22–0.78
	after X-ray	0.74	0.62–0.85	0.73	0.59–0.87	0.94	0.82–1.00

FRAX (Hip) with BMD	before X-ray	0.53	0.37–0.69	0.60	0.42–0.79	0.64	0.18–1.00
	after X-ray	0.66	0.52–0.80	0.63	0.47–0.79	0.74	0.39–1.00

FRAX (Osteo) with BMD	before X-ray	0.54	0.38–0.69	0.62	0.43–0.82	0.69	0.30–1.00
	after X-ray	0.72	0.60–0.84	0.70	0.55–0.84	0.95	0.84–1.00

ROC: receiver operating characteristic; AUC: area under the curve; CI: confidence interval.

BMD: Bone mineral density; Osteo: osteoporotic.
