# Corrigendum to “Predictors of Fracture Risk and Bone Mineral Density in Men with Prostate Cancer on Androgen Deprivation Therapy”

**DOI:** 10.1155/2017/4982312

**Published:** 2017-07-12

**Authors:** Katherine Neubecker, Beverley Adams-Huet, Irfan M. Farukhi, Rosinda Castanon, Ugis Gruntmanis

**Affiliations:** ^1^Department of Medicine, University of Texas, Southwestern Medical Center, Dallas, TX 75390, USA; ^2^Departments of Clinical Sciences and Medicine, University of Texas, Southwestern Medical Center, Dallas, TX 75390, USA; ^3^Department of Nuclear Medicine, Dallas Veterans Affairs Medical Center, Dallas, TX 75216, USA; ^4^Department of Medicine, Dallas Veterans Affairs Medical Center and University of Texas, Southwestern Medical Center, Dallas, TX 75216, USA

In the article titled “Predictors of Fracture Risk and Bone Mineral Density in Men with Prostate Cancer on Androgen Deprivation Therapy” [1], there was an error regarding the FRAX® tool, which should be clarified as follows.

The article notes, “Fracture Risk Assessment (FRAX) is an algorithm developed by the World Health Organization to determine fracture risk of men and women [8].” However, the World Health Organization (WHO) did not develop, test, or endorse the FRAX tool or its recommendations [2]. The metabolic bone disease unit at the University of Sheffield that developed FRAX was a WHO Collaborating Centre from 1991 to 2010, but treatment guidelines must undergo a formal process before they can be endorsed by the WHO.
